# Crystal structure and mutation analysis revealed that DREP2 CIDE forms a filament-like structure with features differing from those of DREP4 CIDE

**DOI:** 10.1038/s41598-018-36253-y

**Published:** 2018-12-13

**Authors:** Hyun Ji Ha, Hyun Ho Park

**Affiliations:** College of Pharmacy, Chung-Ang University, Seoul, 06974 Republic of Korea

## Abstract

Cell death-inducing DFF45-like effect (CIDE) domain-containing proteins, DFF40, DFF45, CIDE-A, CIDE-B, and FSP27, play important roles in apoptotic DNA fragmentation and lipid homeostasis. The function of DFF40/45 in apoptotic DNA fragmentation is mediated by CIDE domain filament formation. Although our recent structural study of DREP4 CIDE revealed the first filament-like structure of the CIDE domain and its functional importance, the filament structure of DREP2 CIDE is unclear because this structure was not helical in the asymmetric unit. In this study, we present the crystal structure and mutagenesis analysis of the DREP2 CIDE mutant, which confirmed that DREP2 CIDE also forms a filament-like structure with features differing from those of DREP4 CIDE.

## Introduction

The activation of endonucleases with cleavage of chromosomal DNA into ~180-base pair fragments is one of the main features of apoptotic cells^1–3^. Among the several identified endonucleases, including apoptosis-inducing factor (AIF) and EndoG, DNA fragmentation factor 40/45 (DFF40/45) is considered as the main player in the process of DNA fragmentation during apoptosis. Additionally, AIF and EndoG were shown to be involved in initial large-scale DNA fragmentation during apoptosis, while the DFF40/45 hetero-dimeric complex is involved in further small-scale cleavage^1,2,4,5^. DFF40 is an endonuclease directly involved in the DNA cleavage process, while DFF45 is an inhibitor that suppresses the nuclease activity of DFF40 under normal conditions via a tight interaction^3,6^. During the apoptosis signalling process, activated effector caspases, such as caspase-3, cleave DFF45, allow DFF40 to dissociate from DFF45, enter the nucleus, and degrade chromosomal DNA^3,7,8^.

Both DFF40 and DFF45 have a common protein interacting module known as the cell death-inducing DFF45-like effect domain (CIDE domain), which is approximately 90 amino acid residues^2,9^. According to the sequence homology search, CIDE domain-containing proteins such as CIDE-A, CIDE-B, and CIDE-3 (FSP27 in mouse) have been discovered in human genome^10,11^. Although the main function of CIDE domain-containing proteins was a regulator of apoptosis, several knock-out mouse studies revealed that they do function on lipid metabolism by localizing to lipid droplets and the endoplasmic reticulum^12–15^.

Apoptotic DNA fragmentation and lipid metabolism are conserved in *Drosophila Melanogaster*, and DREP1, DREP2, DREP3, and DREP4 have been identified as CIDE domain-containing proteins in fly^16,17^. DREP1 and DREP4 are homologues of DFF45 and DFF40, respectively. DREP2 has been suggested as another endonuclease that is inhibited by DREP3 through interactions with CIDE domains^18–20^. A recent biochemical study showed that DREP2 interacts with both DREP1 and DREP3 via the CIDE domain^17,21^. Novel functions for DREP2 in learning and memory in fly brain synapses have been suggested^22^. However, the function and biological implications of the interactions of DREP2 with their binding partners in the cell are unknown.

CIDE domain-mediated protein interactions have been investigated in structural studies of several CIDE domains and hetero-dimeric and homo-dimeric complex structures^23–29^. We recently reported the filament-like CIDE domain assembly of DFF40 and DREP4 and its functional implications in the apoptotic DNA fragmentation process^30^. In the initial structural study of CIDE domain assembly, we solved the structure of the DREP2 CIDE domain. However, this structure was not helical in the asymmetric unit, making it unclear how these proteins assemble into highly oligomeric forms in solution. Based on the structural study of DREP4 CIDE, which showed a helical filament-like structure even in the crystallographic asymmetric unit, we found that the CIDE domains of both DREP4 and DREP2 form filament-like structures in solution. In this study, the details of the helical assembly of the CIDE domain were determined and the filament-like helical oligomeric complex of DREP2 CIDE was confirmed by structural analysis and mutagenesis studies. Additionally, the differences in the characteristics of filament-like structures between DREP2 and DREP4 were determined.

## Results

### CIDE domain of DREP2 forms a highly oligomeric state in solution

**Figure 1:**
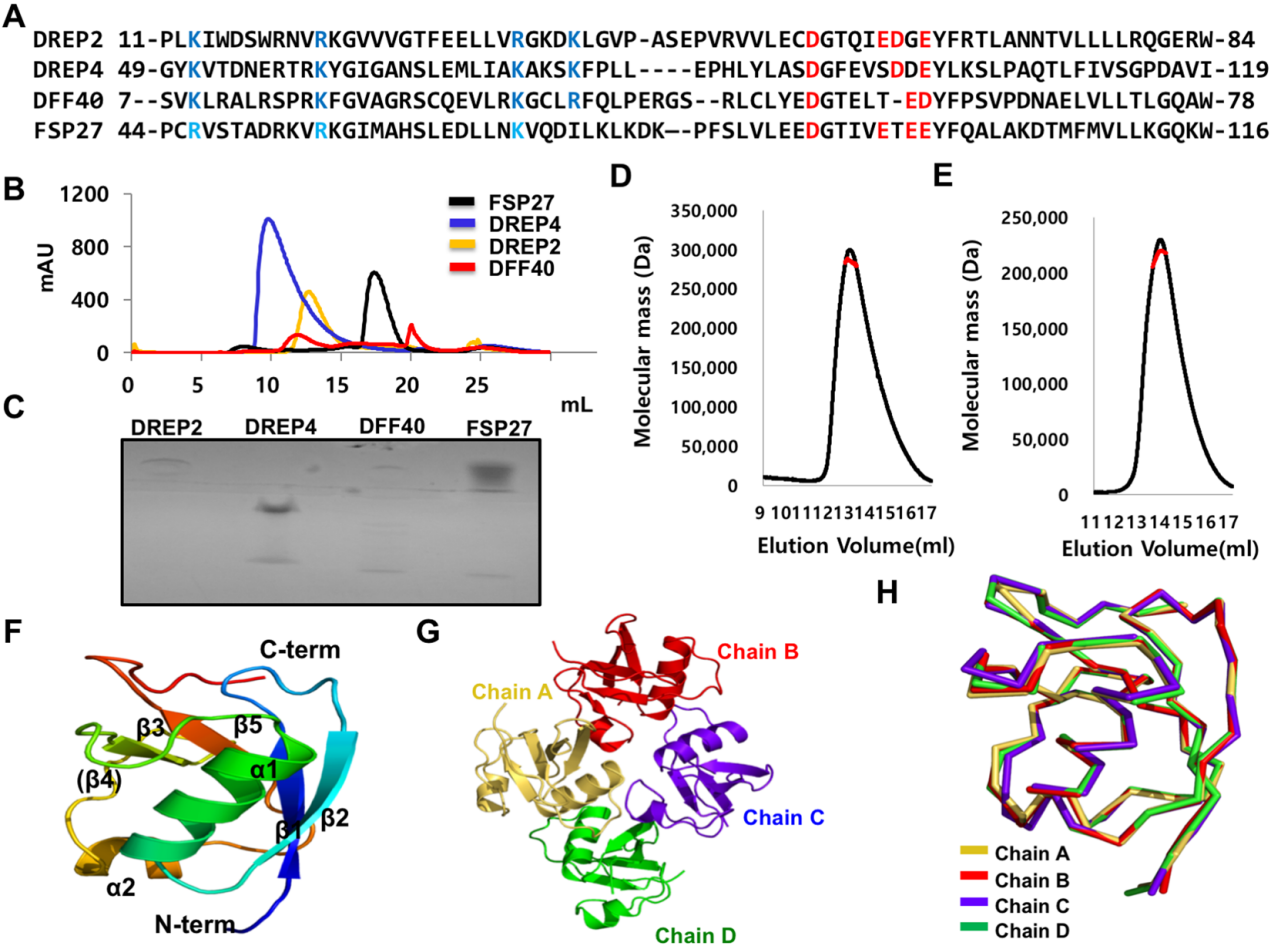
Structure of DREP2 CIDE domain. (**A**) Sequence alignment of CIDE domains. Residues conserved and involved in hetero-dimeric and homo-dimeric complexes are coloured as blue for basic residues and red for acidic residues. (**B**) Size-exclusion chromatograms of the DREP2 CIDE domain and other CIDE domains. The profile obtained from DREP2 CIDE is indicated by a black line, DREP4 CIDE as a blue line, FSP27 CIDE as a yellow line, and DFF40 CIDE as a red line. All size-exclusion chromatography experiments were performed using 20 mM Tris-HCl and 150 mM NaCl buffer. (**C**) Native-PAGE with CIDE proteins. (**D**) and (**E**). Multi-angle light scattering (MALS) measurement of the DREP2 CIDE domain in 50 mM NaCl (**D**) and 1 M NaCl (**E**) showing the absolute molecular mass of the protein. The x-axis and y-axis indicate the elution volume and molecular mass, respectively. (**F**) Ribbon diagram of monomeric DREP2 CIDE domain. The chain from the N- to the C-termini is coloured as blue to red. Helices and sheets are labelled. (**G**) Ribbon diagram of the oligomeric DREP2 CIDE domain found in the asymmetric unit. Chains A–D are shown separately. (**H**) Superposition of each chain of the oligomeric CIDE domain.

Apoptotic DNA fragmentation, which is mainly mediated by the DFF40/DFF45 heterocomplex, is the hallmark of apoptotic cell death and conserved in fly. Four CIDE domain-containing proteins have been identified in fly: DREP1, DREP2, DREP3, and DREP4. Unlike DFF45, which contains only conserved acidic residues, the DREP2, DREP4, DFF40, and FSP27 CIDE domains contain two patches, acidic and basic, in one CIDE domain (Fig. 1A). Although the pattern of charge distribution of each CIDE domain is similar in that they contain two oppositely charged patches, their behaviours differed in solution by forming various oligomeric states based on the size-exclusion chromatography results (Fig. 1B). The DREP4 CIDE domain showed the greatest oligomerization in solution (Fig. 1B). In size-exclusion chromatography, the DREP2 CIDE domain was eluted at approximately 11–14 mL, corresponding to a molecular weight of 100–300 kDa, indicating formation of a highly oligomeric complex (Fig. 1B). We also performed native-PAGE with purified DREP2, DREP4, DFF40, and FSP27 proteins to check the behaviour of the CIDE domain in native state. As shown by the Fig. 1C, we found that DREP2 and DFF40 were stuck on the loading wall and did not migrate well on the native-gel, which might be due to large size of the homo-oligomeric structure. DREP4 migrated a little on the gel. This is also due to the large size of the DREP4 CIDE as indicated by size-exclusion chromatography. Interestingly, however, majority of the FSP27 CIDE, which was eluted as 18 mL on the size-exclusion chromatography, did not migrated well on the gel, although some low molecular weight particle was shown on the gel. This might be because FSP27 also formed higher oligomeric filament structure during the concentration process.

Oligomerization of the CIDE domain of DREP4 was highly dependent on the concentration of salt^30^. To analyse the salt dependency of complex formation of DREP2, we conducted multi-angle light scattering (MALS), which reveals the absolute molecular weight of a particle. The molecular weight of DREP2 CIDE in 50 mM NaCl and 1 M NaCl were calculated by MALS. The theoretical molecular weight of DREP2 CIDE was 10.99 kDa, while the experimental weights determined by MALS were 281.6 kDa (2.04% fitting error) in 50 mM NaCl and 215.3 kDa (0.77% fitting error) in 1 M NaCl, indicating that complex formation of DREP2 CIDE is not dependent on the salt concentration (Fig. 1D,E). Oligomerization of the CIDE domain was expected, as several studies showed that CIDE domain-containing proteins formed highly oligomeric complexes via this domain^31^.

### Structure of DREP2 CIDE

The 2.3 Å high resolution crystal structure of the DREP2 CIDE domain was solved using the molecular replacement (MR) method followed by refining to an R_work_ of 22.0% and R_free_ of 25.6%. The structure of the DREP2 CIDE domain exhibited an atypical CIDE domain fold, which is composed of an α/β roll fold with two α helices and five β strands, containing the α2 helix, but not β4 strand (Fig. 1F). This case is similar with the structure of ICAD CIDE and CIDE-A. The two helices comprised of residues 30–41 and 64–68 are indicated as α1 and α2, respectively. The four strands comprised of residues 10–15, 22–27, 50–53, and 74–78 are indicated as β1, β2, β3, and β5, respectively (Fig. 1F). Unlike DREP4 CIDE, in which one turn of the helical complex is formed by 10 molecules of DREP4 CIDE in the crystallographic asymmetric unit, there were four molecules in the asymmetric unit of DREP2 CIDE, which were referred to as chains A–D (Fig. 1G). A model of chains A and D was built from residues 7–84, while that of chains B and C was built from residues 8–84. There were extra residues of Leu, Glu, and His at the C-terminus, which were part of the vector construct. Based on the Ramachandran plot, 95% of the residues were in the most favourable region, whereas 5% were in the allowed regions. The collection and processing of data and refinement statistics are summarized in Table 1. Interestingly, there was no apparent symmetry between the four chains (Fig. 1G). Each monomer was nearly identical, as indicated by superimposition with a root mean square deviation (R.M.S.D.) of approximately 0.7–0.9 Å (Fig. 1H).

**Table 1:** Crystallographic statistics.

Data collection	Wild-type	R36E
Space group	*P*2_1_2_1_2_1_	*P*2_1_2_1_2_1_
Cell dimensions		
*a*, *b*, *c*	50.3 Å, 88.7 Å, 113.4 Å	49.9 Å, 88.6 Å, 113.4 Å
Resolution	50–2.3 Å	50–3.3 Å
^†^*R*_sym_	5.6% (28.4%)	18.5% (51.7%)
^†^Mean I/σ (I)	55.8 (5.2)	13.8 (4.6)
^†^Completeness	95.4% (98.3%)	99.9% (98.7%)
^†^Redundancy	9.7 (5.9)	7.0 (7.0)
**Refinement**		
Resolution	33–2.3 Å	43–3.3 Å
No. of reflections used	24,774	7,935
*R*_work_/*R*_free_	22.0%/25.6%	18.0%/23.4%
No. atoms		
Protein	2602	2590
Water and other small molecules	38	0
Average B-factors		
Protein	53.6 Å^2^	52.4 Å^2^
Water	44.2 Å^2^	
R.M.S. deviations		
Bond lengths	0.009 Å	0.014 Å
Bond angles	1.381°	1.361°
Ramachandran plot		
Most favoured regions	95.8%	94.9%
Additional allowed regions	4.2%	5.1%

^†^Highest resolution shell is shown in parentheses.

### Four interfaces are formed in the oligomeric CIDE domain of DREP2

**Figure 2:**
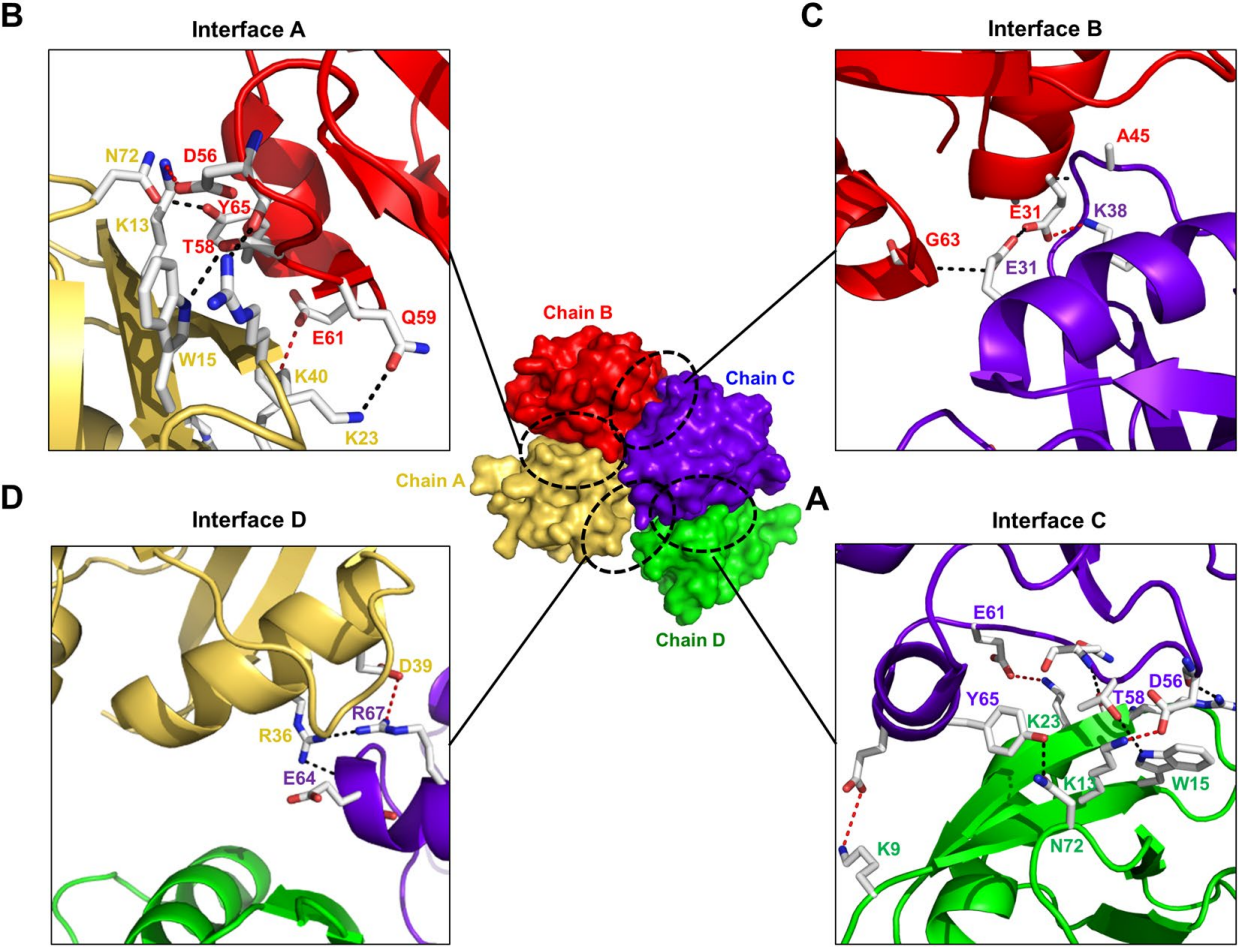
Tetrameric interface of the structure of the DREP2 CIDE domain. The tetrameric structure is shown in the centre of the figure. Close-up views of the interacting residues in the interface between chain C and chain D (**A**), chain A and chain B (**B**), chain B and chain C (**C**), and chain D and chain A (**D**) are shown on the corner of each side. The residues involved in the contact are shown. Salt bridges formed between one chain and its counterpart are shown as red-dashed lines. H-bonds are shown as black-dashed lines.

The four CIDE domains in the asymmetric unit are arranged to form a square-shaped complex through non-symmetric interactions between molecules, resulting in mediation of the tetrameric arrangement by four unique interfaces involving different parts of the surface and residues of the DREP2 CIDE domain. The interface between chains C and D (hereafter, interface C) showed the most extensive interactions burying the 500-Å^2^ accessible surface area. Inspection of interface C revealed that the main interactions are mediated by polar residues involving K9, K13, W15, R22, K23, and N72 from chain D and D56, T58, Q59, E61, E64, and Y65 from chain C. These residues form three salts bridges and four hydrogen bonds, as well as contribute to van der Waals interactions (Fig. 2A). The second largest interface, which is formed by chains A and B (hereafter, interface A), buries the 495-Å^2^ accessible surface area and is mediated by the same residues observed in interface C, except for residues K9 (chain D) and E64 (chain C) (Fig. 2B). Interestingly, these two interfaces (A and C) are highly similar to those observed in the homo-dimeric and hetero-dimeric CIDE domain complex^23,26,28,29^. These two CIDE homo-dimers further assembled into a tetrameric complex by forming two unique interfaces between chains B and C (hereafter, interface B) and between chains A and C (hereafter, interface D). Interface B buries the 368-Å^2^ accessible surface area, and the interaction is mediated by residues E31, T29, and G61 from chain B and E31, K38, and A45 from chain C (Fig. 2C). The last interface, D, buries the 134-Å^2^ accessible surface area via an interaction mediated by residues E61, D62, and R67 from chain C and residues E36, D39, and K40 from chain A (Fig. 2D). In the tetrameric assembly, chain C forms three unique interfaces with chains A, B, and D involving a surface area of 1002 Å^2^ which accounts for 20% of the total surface area of 4951 Å^2^ of chain C, while chain D only forms an interface with chain C. Therefore, the chain C molecule is important in the tetrameric arrangement of the DREP2 CIDE domain in the asymmetric unit.

### Putative oligomeric structure of CIDE domain of DREP2

**Figure 3:**
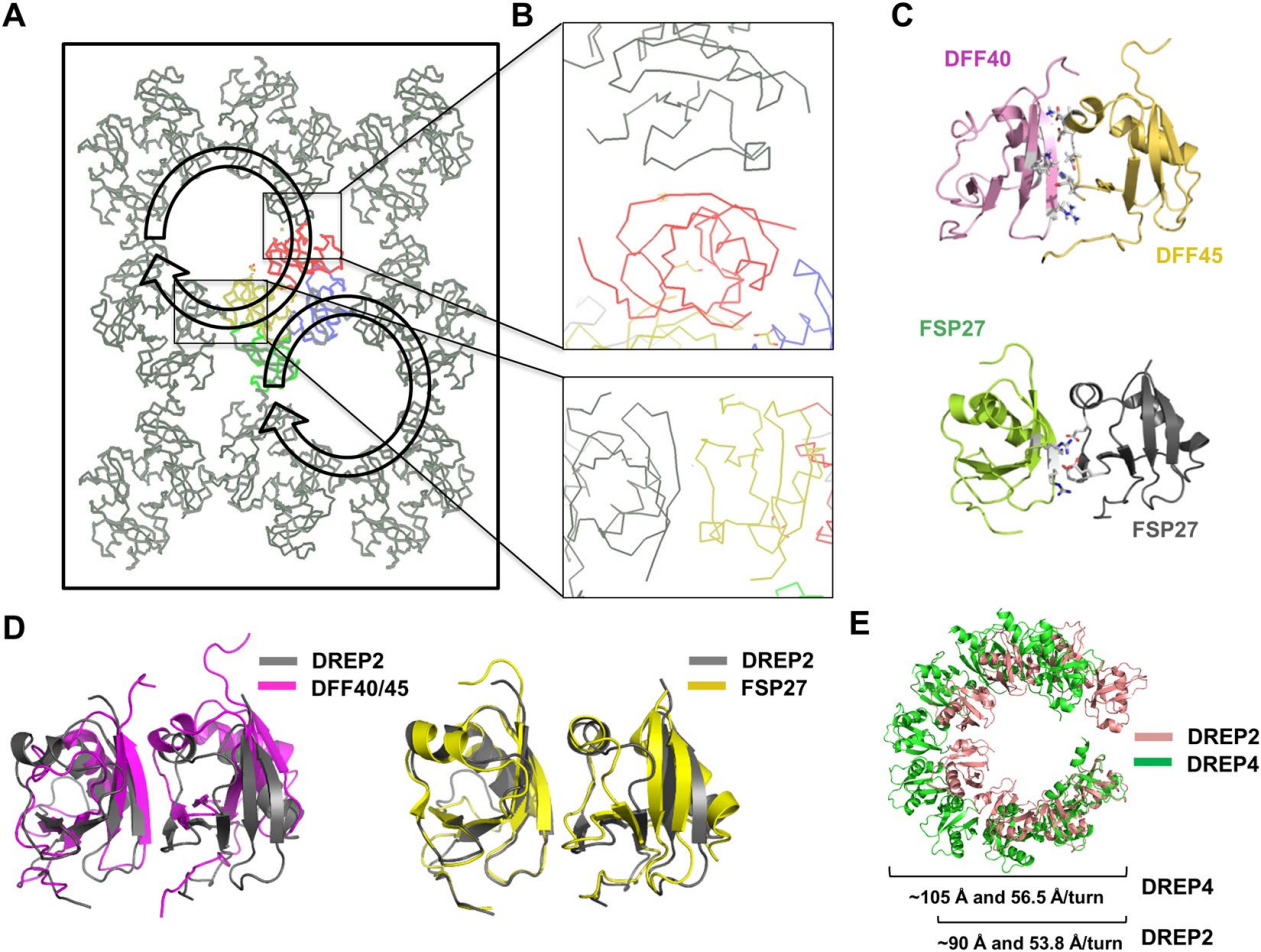
Putative oligomeric structure of the CIDE domain of DREP2. (**A**) Schematic drawing of the crystallographic packing of the DREP2 CIDE domain. The colour unit indicates the tetrameric structure detected in the asymmetric unit of the crystal. (**B**) Close-up view of the interface formed between chain B and the neighbouring CIDE domain from crystallographic packing (upper panel). Close-up view of the interface formed between chain A and neighbouring CIDE domain from crystallographic packing (lower panel). (**C**) Structure of DFF40 CIDE: DFF45 CIDE complex, which is a representative hetero-dimeric complex of CIDE domains. The residues on the basic-charged surface of DFF40 interact with those on the acidic-charged surface of DFF45 (upper panel). Structure of the homo-dimeric complex of the FSP27 CIDE domain. Residues on the basic-charged surface of FSP27 interact with the residues on the acidic-charged surface of another FSP27 (lower panel). (**D**) Superposition of dimeric DREP2 with hetero-dimeric DFF40/45 (left panel) and homo-dimeric FSP27 (right panel). (**E**) Comparison of tentative filament-like structure of eight molecules of DREP2 with filament-like structure formed by 10 molecules of DREP4.

The DREP2 CIDE domain exists as a highly oligomeric complex in solution, containing 16–18 molecules in the complex as determined by size-exclusion chromatography and MALS. Because the current tetrameric structure of the DREP2 CIDE domain is smaller than the calculated size, we examined the entire molecular packing of the CIDE domains in the crystal (Fig. 3A). Interestingly, each tetrameric complex of the DREP2 CIDE domain, which was detected in the asymmetric unit, was linked to the next tetrameric unit using interfaces previously detected in the hetero-dimeric and homo-dimeric complexes of CIDE domains (Fig. 3B)^26,29^. The solution structure of the hetero-dimeric complex between DFF40 CIDE and DFF45 CIDE showed that the interaction is mediated by a basic patch (K9, K18, K32, and R36) on DFF40 CIDE and acidic patch (D66, D71, D72, and D74) on DFF45 CIDE^27^ (Fig. 3C). Another structural study of the homo-dimer of the FSP27 CIDE domain revealed that homo-dimerization of the CIDE domain is mediated by basic patch formed by R46, R55, and K56 of one FSP27 CIDE and acidic patch formed by E87, D88, and E93 of the second CIDE (Fig. 3C). This interaction strategy may be the same as that used by neighbouring molecules in the structure of DREP2 CIDE. Superposition of the structures showed that the hetero-dimeric DFF40/DFF45 CIDE domain complex and homo-dimeric FSP27 CIDE domain were well-superimposed with the homo-dimeric structure of the DREP2 CIDE domain, which was formed by one CIDE domain from a real tetrameric complex and another from crystallographic packing with R.M.S.D. values of 2.8 and 2.6 Å, respectively (Fig. 3D). These findings indicate that the orientation of the interface formed by crystallographic packing was similar to that formed by the hetero-dimeric complex and homo-dimeric complex of the CIDE domains. This structural analysis and the newly solved filament-like helical structure of DREP4 CIDE suggest that DREP2 CIDE forms a similar filament-like helical structure in solution. Therefore, we modelled the helical complex of DREP2 by identifying the symmetrical molecules responsible for forming the helical complex in the crystal lattice (Fig. 3A,E). Our previous structural study of the filament-like CIDE domain showed that 10 molecules of DREP4 CIDE form one turn of the helical assembly in the crystallographic asymmetric unit^30^. In the crystal lattice, the helical structure is continuous and stacks along the a-axis of the unit cell with a 56.5 Å rise/turn and ~105 Å diameter^30^. The modelled helical structure of DREP2 based on the crystal contains four molecules in the crystallographic asymmetric unit arranged into a filament assembly with eight subunits per turn, rise of 50.3 Å, and diameter of ∼90 Å (Fig. 3E).

### Confirmation of helical complex of DREP2 by mutagenesis

**Figure 4:**
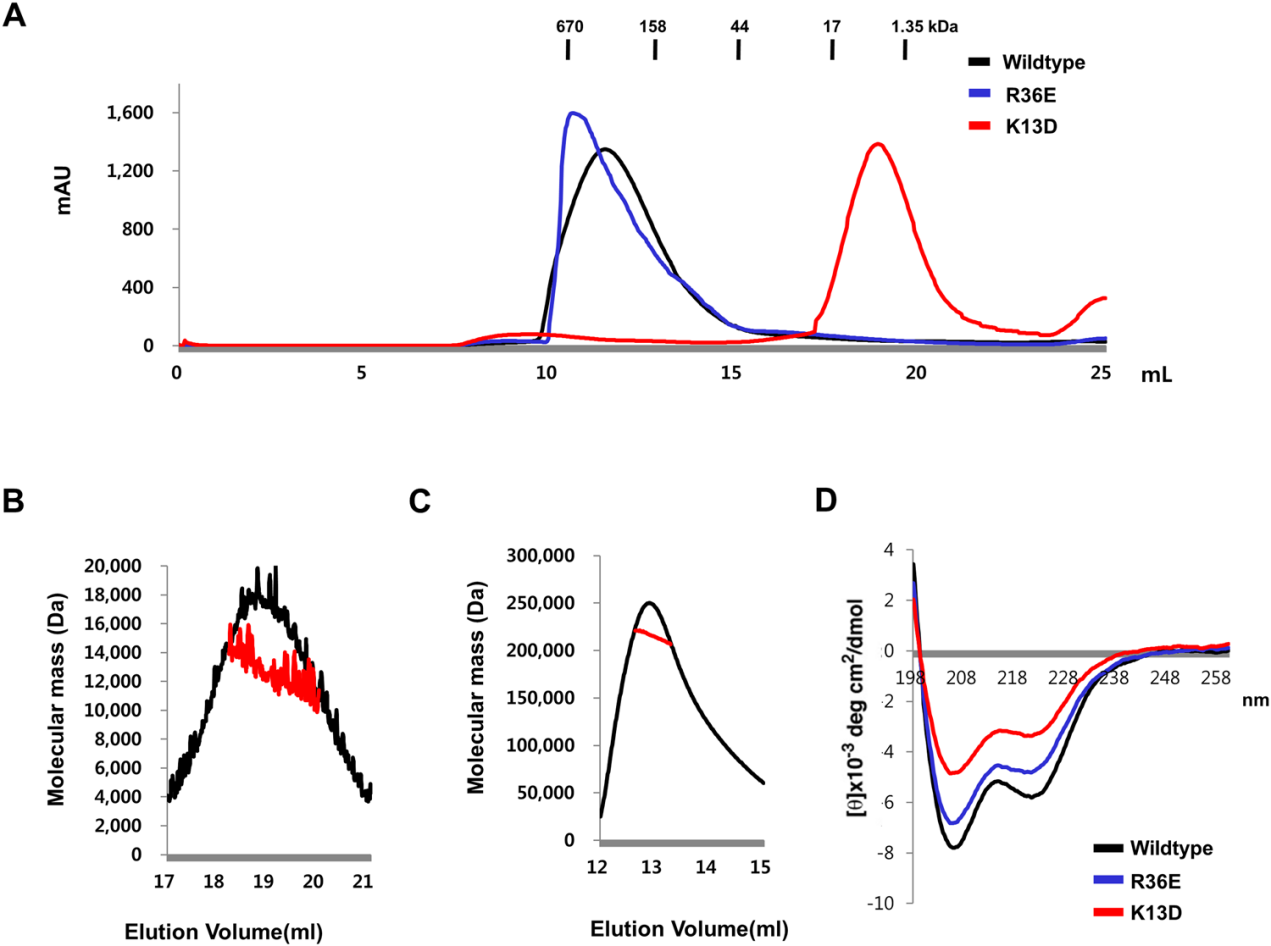
Mutation that disrupts the interface formed in crystallographic packing, K13D, disrupted the oligomeric state of the DREP2 CIDE domain. (**A**) Size-exclusion chromatograms of DREP2 CIDE and its mutants. The profile obtained from the wild-type DREP2 CIDE domain is indicated with a black line, K13D mutant with a red line, and R36E mutant with a blue line. All size-exclusion chromatography experiments were performed using 20 mM Tris-HCl and 150 mM NaCl buffer. (**B** and **C**) Multi-angle light scattering (MALS) measurement of the K13D (**B**) and R36E (**C**) showing the absolute molecular mass of the protein. The x-axis and y-axis indicate the elution volume and molecular mass respectively. (**D**) Circular dichroic spectra of wild-type DREP2 CIDE (black line), K13D mutant (red line), and R36E (blue line). The spectra were recorded at 25 °C, and four scans were conducted and averaged using a J-715 spectropolarimeter (Jasco).

To determine the importance of the interface formed by crystallographic packing in the oligomeric complex followed by formation of further filament-like structures, we conducted a mutagenesis study. Based on interface analysis of the tetrameric DREP2 and previous analysis of the interface formed by the hetero-dimeric DFF40/DFF45 CIDE complex showing that the main forces generated in the interface were salt bridges formed by K9, K18, K32, and R36 on DFF40 and D66, D71, D72, and D74 on DFF45, we introduced a mutation at K13 on the DREP2 CIDE domain to D (hereafter, K13D), which is aligned with K9 in DFF40, to disrupt the interfaces A and B in the tetrameric DREP2 structure. We also introduced a mutation at R36 in the DREP2 CIDE domain to E (hereafter, R36E), which is critical for the formation of interface D in the tetrameric DREP2 structure (Fig. 2D). While wild-type DREP2 CIDE and R36E mutants formed a highly oligomeric homo-complex in solution that was eluted from the gel-filtration column at approximately 11 mL, the K13D mutant produced a monomeric peak in solution that was eluted from the gel-filtration column at approximately 18 mL, indicating that only K13D (disrupting interfaces A and B) disrupted complex formation (Fig. 4A). The molecular weight of the disrupted K13D mutant was confirmed by MALS. The theoretical molecular weight of the K13D mutant was 10.85 kDa and the experimental molecular weight determined by MALS was 12.14 kDa (8.59% fitting error), with a polydispersity of 1.000 (Fig. 4B). MALS showed that R36E still formed a ~217.4 kDa complex, with a similar size as the wild-type (Fig. 4C). To exclude the possibility that dissociation of the self-complex resulted from structural distortion caused by mutations, we conducted far UV circular dichroism (CD) analysis. As shown in Fig. 4D, the wild-type and two mutants showed similar CD spectrum patterns, with two pronounced minima at 208 and 222 nm and a maximum at 215 nm. These findings indicate that the mutations did not affect the DREP2 CIDE domain structure. Accordingly, the real form of DREP2 CIDE in solution is a helical filament-like complex rather than a tetrameric complex which is packed and detected in the asymmetric unit.

**Figure 5:**
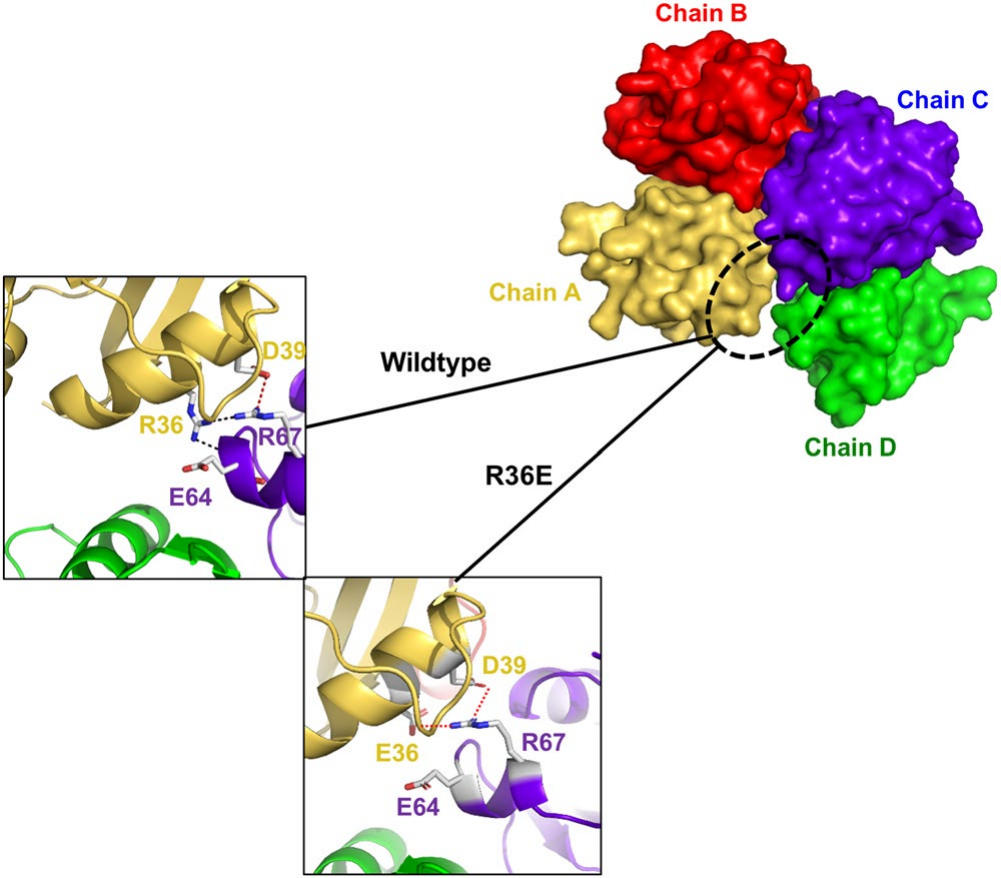
Structure of R36E mutant of DREP2 CIDE domain. Surface figures of oligomeric R36E DREP2 CIDE mutant found in the asymmetric unit, Chains A–D are shown in the middle. Mutated area, R36E, located in the interface formed in between chains A and D is shown and compared with that of wild-type (two individual boxes).

The R36E mutant was crystallized under similar conditions as those used to produce the wild-type crystal. The structure of R36E was solved and refined to an R_work_ of 18.0% and R_free_ of 23.4%. Four molecules in the asymmetric unit were the same as those in the wild-type (Fig. 5). Mutated E36 formed a salt bridge with R67 from neighbouring molecule chain C (Fig. 5). This interface was formed as a crystallographic artefact, which was confirmed by mutagenesis analysis.

### Characterization of DREP2 CIDE and comparison with DREP4 CIDE

**Figure 6:**
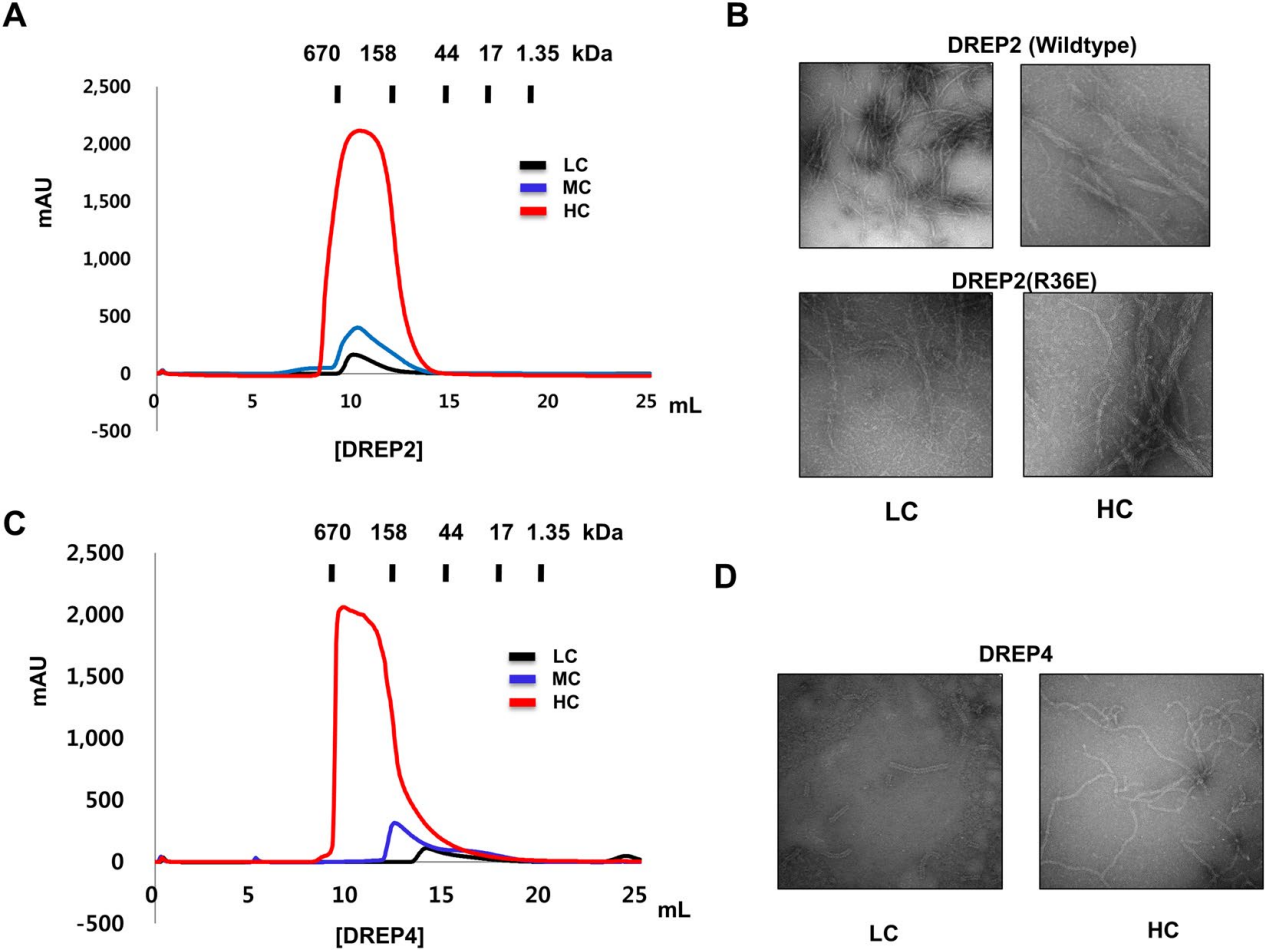
Comparison of oligomerization states between DREP2 and DREP4. (**A**) Size-exclusion chromatograms of DREP2 CIDE in three different concentrations, 30 mg/mL (high concentration: LC), 5 mg/mL (middle concentration: MD), and 1 mg/mL (low concentration: LC). DREP2 CIDE formed large oligomers in both high and low concentration. (**B**) Electron microscopy images of negatively stained wild-type DREP2 CIDE and R36E mutant, showing rings and filaments in both high (HC) and low (LC) concentration. (**C**) Size-exclusion chromatograms of DREP4 CIDE in three different concentrations. DREP4 CIDE formed large oligomers at a high concentration and migrated at smaller sizes with deceasing the sample concertation. (**D**) Electron microscopy images of negatively stained DREP4 CIDE, showing much longer filaments at the high concentration (HC) but smaller filaments at the low concentration (LC).

Previous structural analysis showed that the CIDE domain forms helical oligomers through repetitive head-to-tail polymerization via charged interfaces, which are disrupted by a high concentration of NaCl^30^. As the salt concentration was increased, the DREP4 CIDE particle sizes decreased. Because the particle size of the CIDE domain varied depending on the buffer condition, protein concentration-dependent oligomerization, which is another important factor affecting filament formation, was also examined by size-exclusion chromatography and electron microscopy. The size of the DREP2 CIDE particle did not change as protein concentration was decreased from 30 to 1 mg/mL (Fig. 6A). Electron microscopy of negatively stained samples also showed that both DREP2 and the R36E mutant formed similar-sized rings and filaments in solution and that the filament size was not affected by protein concentration (Fig. 6B). In contrast, filament formation of DREP4 CIDE was dependent on the protein concentration based on the results of size-exclusion chromatography (Fig. 6C) and electron microscopy (Fig. 6D). Smaller particles eluted at 15 mL in the size-exclusion chromatography (Fig. 6C) and smaller filaments were detected by electron microscopy (Fig. 6D) when low concentrations of DREP4 CIDE were used, strongly indicating that the filament formation of DREP4 CIDE depends on the protein concentration, whereas the DREP2 CIDE filament is not sensitive to protein concentration. This indicates that each CIDE domain exhibits unique features in filament formation, although the structure and surface features are identical.

## Discussion

Apoptotic DNA fragmentation, which is a hallmark of apoptosis, is mediated by apoptotic nuclease DFF40. DFF40 contains a CIDE domain, which is a protein interaction domain. The CIDE domain-mediated interaction of DFF40 with DFF45, an inhibitor of DFF40 that contains a CIDE domain, is critical for controlling the activity of DFF40. Filament-like assembly of DFF40 via CIDE domain after removing DFF45 upon activation of apoptotic signalling is necessary for DNA fragmentation. In addition to its function in DNA fragmentation, the CIDE domain-containing proteins CIDEA, CIDEB, and FSP27 play important roles in lipid homeostasis. It has been reported that CIDEA, CIDEB, and FSP27 localize at lipid droplet contact sites, promoting lipid transfer and lipid droplet fusion in adipocytes and hepatocytes. Because of this involvement, CIDE-containing proteins were emerged targets for therapeutic intervention of metabolic disorders.

**Figure 7:**
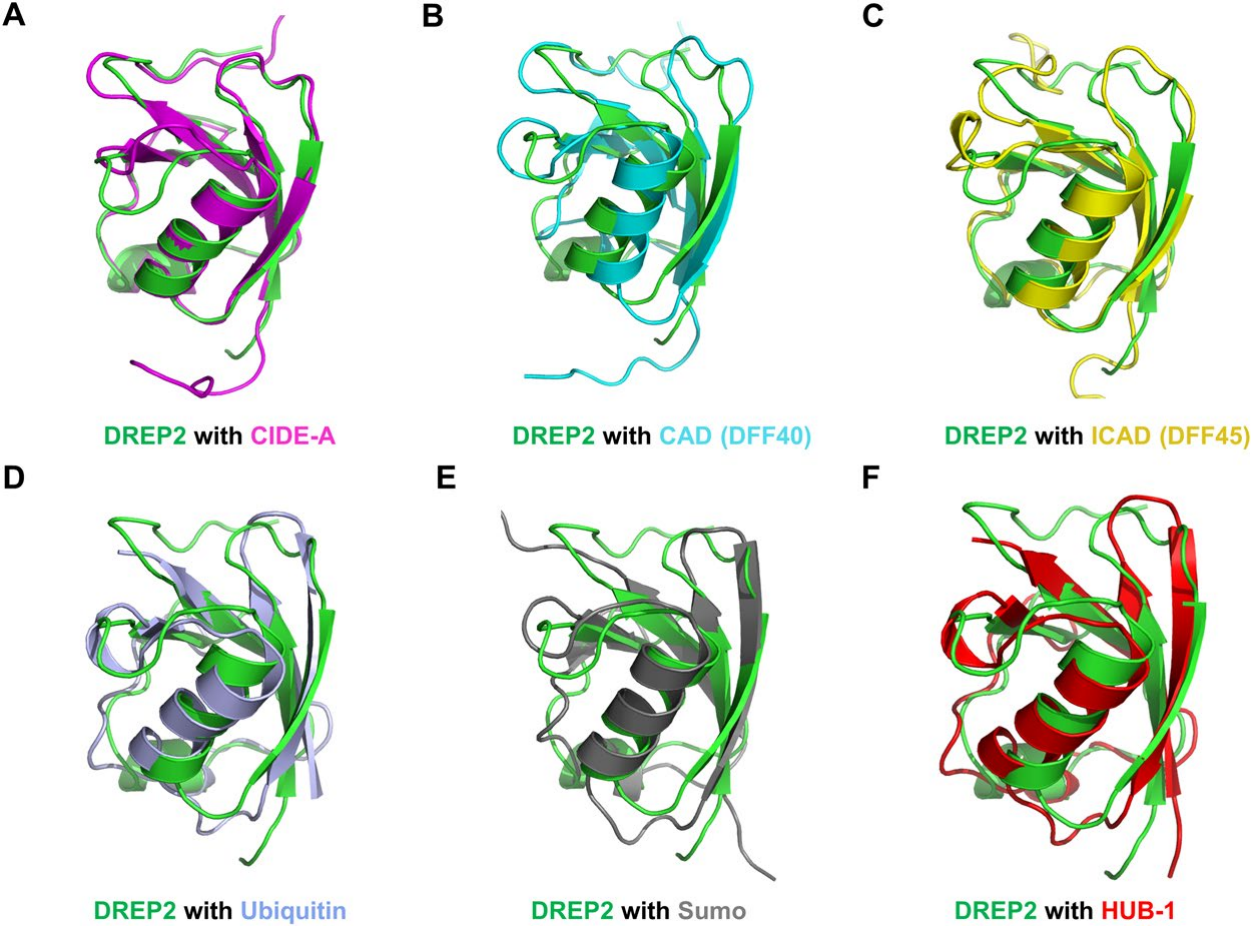
Superposition of the DREP2 CIDE domain with its structural homologues. The monomeric DREP2 CIDE domain and six structural homologues are superimposed pairwise. The DREP2 CIDE domain is green and its counterparts are magenta for CIDE-A (**A**), cyan for CAD (**B**), yellow for ICAD (**C**), light blue for ubiquitin (**D**), grey for Sumo (**E**), and HUB-1 for red (**F**).

The high resolution structure of the DREP2 CIDE showed that the structure exhibited an atypical CIDE structure that contains two helices, α1 and α2, and four strands, β1, β2, β3, and β5, by replacing β4 with a loop. A structural homology search conducted using DALI^32^ revealed that the DREP2 CIDE domain is highly similar to ubiquitin-like domains and other CIDE domains (Table 2). DALI server picked six structures, including CIDE-A (PDBid: 2EEL), CAD (PDBid: 1F2R-I), ICAD (PDBid: 1F2R-C), ubiquitin (PDBid: 4NQK-E), SUMO-3 (PDBid: 1U4A-A), and HUB-1 (PDBid: 3PLU-A), as top matches. Pair-wise structural alignments of the DREP2 CIDE domain and structural homologues showed that the length and orientation of the α2 helices in the DREP2 CIDE domain differed slightly from those of in the other structures (Fig. 7A–F). A district β4 was only detected in the structure of CAD (Fig. 7B). No ubiquitin-related proteins contained β4 (Fig. 7D–F). One of the distinct features of ubiquitin-related proteins, including ubiquitin and Sumo, is that β2 is longer than the CIDE domain (Fig. 7A–F). Based on the structural similarity between the CIDE domain and ubiquitin, it would be interesting to functionally characterize and compare these domains.

**Table 2:** Structural similarity search using DALI.

Proteins and accession numbers	Z-score	RMSD (Å)	Identity (%)	References
CIDE-A (2EEL:A)	9.1	1.10	33	Not published
CAD (1F2R:I)	8.7	1.65	26	^26^
ICAD (1F2R:C)	8.2	2.18	32	^26^
Ubiquitin (4NQK:E)	7.7	1.88	22	^40^
Sumo-3 (1U4A:A)	7.8	1.97	16	^41^
HUB-1 (3PLU:A)	7.3	2.00	12	^42^

Although the structure of DREP2 CIDE is similar to those of other CIDEs in that it contains a typical α/β roll fold with two α helices and five (or four) β strands, the high-resolution structure revealed a possible biologically important higher oligomerization mechanism of the CIDE domain that functions through several novel dimeric interfaces formed between homo-dimers. Before solving helical filament-like oligomeric structure of DREP4, determining the DREP2 structure, which formed an unusual tetramer without any symmetry in the crystallographic asymmetry unit, was difficult. The current study showed that DREP2 CIDE also formed a filament-like structure with features differing from those of DREP4 CIDE in solution. The filament-like oligomeric structure of DREP2 was confirmed by mutagenesis analysis.

Although the function of CIDE domain-mediated filament-like structure in apoptotic DNA fragmentation was established in the current structural study, CIDE domain-mediated lipid metabolism and learning/memory in brain synapses require further investigation. The characterization and elucidation of the oligomeric forms of the CIDE domain and CIDE domain-containing proteins will provide important information regarding the function of various CIDE domain-containing proteins in apoptosis, lipid metabolism, and particularly lipid droplet (LD) growth and learning/memory in the brain synapses.

## Methods

### Sequence alignment

Clustal W has been used for analysing the amino acid sequences of CIDEs (http://www.ebi.ac.kr/Tools/clustalw2/index.html).

### Protein expression and purification

The expression and purification methods used in this study have been described in detail elsewhere^21,29,30^. Briefly, the DREP2 CIDE (amino acids 1–84), the DREP4 CIDE (amino acids 39–130), and FSP27 (amino acids 38–119) were expressed in *Escherichia coli* BL21 (DE 3) under overnight induction at 20 °C. The protein contained a carboxyl terminal His-tag and was purified by nickel affinity and size-exclusion chromatography with a S200 gel filtration column 10/30 (GE Healthcare, Little Chalfont, UK) that had been pre-equilibrated with 20 mM Tris-HCl at pH 8.0 and 150 mM or 500 mM NaCl. The protein was then concentrated for further use.

### Native PAGE shift assay

Oligomeric forms of each CIDE domains were monitored by native (non-denaturing) PAGE on a PhastSystem (GE Healthcare) with pre-made 8–25% acrylamide gradient gels (GE Healthcare). Separately purified proteins were directly loaded onto the gel. Coomassie Brilliant Blue was used for staining and detection of the shifted bands. The uncropped scan of gel is provided at Supplementary Fig. 1.

### Crystallization and data collection

The crystallization conditions were initially screened at 20 °C by the hanging drop vapor-diffusion method using various screening kits. Initial crystals were grown on the plates by equilibrating a mixture containing 1 μL of protein solution (7–8 mg/mL protein in 20 mM Tris-HCl at pH 8.0, 500 mM NaCl) and 1 μL of a reservoir solution containing 300 mM magnesium formate dihydrate and 100 mM Bis-Tris pH 6.2 against 0.4 mL of reservoir solution. The best crystal was obtained by further optimization searching over a range of concentrations of protein and precipitant and pH ranges. The diffraction data set was collected at beamline BL-4A of the Pohang Accelerator Laboratory, Republic of Korea. Data processing and scaling were carried out using the HKL2000 package^33^. The mutant crystal was obtained in the similar crystallization condition. 2.3 Å and 3.3 Å data were collected for wildtype and mutant, respectively.

### Structure determination and analysis

The initial molecular-replacement (MR) method was carried out using Phaser^34^ with the solution structure of the CIDE-N domain of human cell death activator CIDE-A (PDB code 2EEL), which has 37% amino-acid sequence identity, as a search model. The MR solution gave rotation-function and translation function Z-scores of 5.4 and 18.9, respectively. Model building and refinement were performed using COOT^35^ and Refmac5^36^, respectively. Model quality was evaluated using PROCHECK^37^. Pymol was used to generate all the cartoon figures^38^.

### Mutagenesis

Site-directed mutagenesis was conducted using a Quick-change kit (Stratagene, La Jolla, CA, USA) according to the manufacturer’s protocols. Mutagenesis was then confirmed by sequencing. Mutant proteins were prepared as described above.

### Oligomerization assay by size-exclusion chromatography

For gel filtration analysis to detect oligomerization formation, the target protein was applied to a gel-filtration column (Superdex 200 HR 10/30, GE Healthcare) that had been pre-equilibrated with 20 mM Tris-HCl 8.0 and 500 mM NaCl. The peak fractions were collected and subjected to SDS-PAGE.

### Multi-angle light scattering (MALS)

The molar mass of the highly oligomerized CIDE domain of DREP2 was determined by MALS. The target protein was injected onto a Superdex 200 HR 10/30 gel filtration column (GE Healthcare). The chromatography system was coupled to a three-angle light scattering detector (mini-DAWN EOS) and refractive index detector (Optilab DSP) (Wyatt Technology, Santa Barbara, CA, USA). Data were collected every 0.5 s at a flow rate of 0.2 mL/min and analysed using the ASTRA program, which gave the molar mass and mass distribution (polydispersity) of the sample.

### Circular dichroism spectroscopy

The secondary structures were measured by circular dichroism (CD) spectroscopy using a J-715 spectropolarimeter (Jasco, Oklahoma City, OK, USA) at the Korea Basic Science Institute in South Korea. The spectra were obtained from 200 to 250 nm at 25 °C in a 0.1-cm path length quartz cuvette at a bandwidth of 1.0 nm, rate of 50 mm/min, and 5-s response time. The protein samples in buffer containing 20 mM Tris-HCl at pH 8.0 and 150 mM NaCl were diluted to 0.1 mg/mL prior to use. Four scans were accumulated and averaged, after which the α-helical content was calculated from the molar ellipticity at 222 nm^39^.

### Electron microscopy

Wild-type DREP2 CIDE and R36E mutant samples after affinity chromatography purification were diluted to 0.8 mg/mL to prepare the high concentration sample and 0.1 mg/mL to prepare the low concentration sample. DREP4 CIDE samples were also diluted to 1 mg/mL as the high concentration sample and 0.1 mg/mL as the low concentration sample. For negative staining, 10 μL of each protein sample was placed onto a glow discharged copper grid and stained with 1% uranyl formate at pH 4.5 for 30 s and air-dried. The grids were imaged using a Tecnai G² Spirit BioTWIN Transmission Electron Microscope (FEI Company, Hillsboro, OR, USA) and recorded with an AMT 2k CCD camera (Thermo Fisher Scientific, Waltham, MA, USA).

### Protein Data Bank accession codes

Coordinates and structural factors have been deposited in the Protein Data Bank.

## Electronic supplementary material

Supplementary Figure

## References

[1] Sakahira H, Iwamatsu A, Nagata S (2000). Specific chaperone-like activity of inhibitor of caspase-activated DNase for caspase-activated DNase. J Biol Chem.

[2] Liu X, Zou H, Slaughter C, Wang X (1997). DFF, a heterodimeric protein that functions downstream of caspase-3 to trigger DNA fragmentation during apoptosis. Cell.

[3] Enari M (1998). A caspase-activated DNase that degrades DNA during apoptosis, and its inhibitor ICAD. Nature.

[4] Susin SA (1999). Molecular characterization of mitochondrial apoptosis-inducing factor. Nature.

[5] Lorenzo HK, Susin SA, Penninger J, Kroemer G (1999). Apoptosis inducing factor (AIF): a phylogenetically old, caspase- independent effector of cell death. Cell Death Differ.

[6] Lechardeur D (2000). Determinants of the nuclear localization of the heterodimeric DNA fragmentation factor (ICAD/CAD). J Cell Biol.

[7] Sakahira H, Enari M, Ohsawa Y, Uchiyama Y, Nagata S (1999). Apoptotic nuclear morphological change without DNA fragmentation. Curr Biol.

[8] Tang D, Kidd VJ (1998). Cleavage of DFF-45/ICAD by multiple caspases is essential for its function during apoptosis. J Biol Chem.

[9] Inohara N, Koseki T, Chen S, Wu X, Nunez G (1998). CIDE, a novel family of cell death activators with homology to the 45 kDa subunit of the DNA fragmentation factor. EMBO J.

[10] Inohara N, Koseki T, Chen S, Benedict MA, Nunez G (1999). Identification of regulatory and catalytic domains in the apoptosis nuclease DFF40/CAD. J Biol Chem.

[11] Liang MC (2003). Jesterone dimer, a synthetic derivative of the fungal metabolite jesterone, blocks activation of transcription factor nuclear factor kappaB by inhibiting the inhibitor of kappaB kinase. Mol Pharmacol.

[12] Nishino N (2008). FSP27 contributes to efficient energy storage in murine white adipocytes by promoting the formation of unilocular lipid droplets. J Clin Invest.

[13] Lin SC, Li P (2004). CIDE-A, a novel link between brown adipose tissue and obesity. Trends Mol Med.

[14] Zhou Z (2003). Cidea-deficient mice have lean phenotype and are resistant to obesity. Nat Genet.

[15] Li JZ (2007). Cideb regulates diet-induced obesity, liver steatosis, and insulin sensitivity by controlling lipogenesis and fatty acid oxidation. Diabetes.

[16] Inohara N, Nunez G (1999). Genes with homology to DFF/CIDEs found in Drosophila melanogaster. Cell Death Differ.

[17] Park OK, Park HH (2013). A putative role of Drep1 in apoptotic DNA fragmentation system in fly is mediated by direct interaction with Drep2 and Drep4. Apoptosis.

[18] Park OK, Park HH (2012). Dual apoptotic DNA fragmentation system in the fly: Drep2 is a novel nuclease of which activity is inhibited by Drep3. FEBS Lett.

[19] Mukae N (2000). Identification and developmental expression of inhibitor of caspase-activated DNase (ICAD) in Drosophila melanogaster. J Biol Chem.

[20] Yokoyama H (2000). A novel activation mechanism of caspase-activated DNase from Drosophila melanogaster. J Biol Chem.

[21] Lee SM, Park HH (2014). *In vitro* analysis of the complete CIDE domain interactions of the Drep system in fly. Apoptosis.

[22] Andlauer, T. F. *et al*. Drep-2 is a novel synaptic protein important for learning and memory. *Elife* **3** (2014). 10.7554/eLife.03895 PMC4229683 25392983

[23] Lee SM, Park HH (2013). General interaction mode of CIDE:CIDE complex revealed by a mutation study of the Drep2 CIDE domain. FEBS Lett.

[24] Fukushima K (2002). Solution structure of the DFF-C domain of DFF45/ICAD. A structural basis for the regulation of apoptotic DNA fragmentation. J Mol Biol.

[25] Lugovskoy AA (1999). Solution structure of the CIDE-N domain of CIDE-B and a model for CIDE-N/CIDE-N interactions in the DNA fragmentation pathway of apoptosis. Cell.

[26] Otomo T, Sakahira H, Uegaki K, Nagata S, Yamazaki T (2000). Structure of the heterodimeric complex between CAD domains of CAD and ICAD. Nat Struct Biol.

[27] Zhou P, Lugovskoy AA, McCarty JS, Li P, Wagner G (2001). Solution structure of DFF40 and DFF45 N-terminal domain complex and mutual chaperone activity of DFF40 and DFF45. Proc Natl Acad Sci USA.

[28] Sun Z (2013). Perilipin1 promotes unilocular lipid droplet formation through the activation of Fsp27 in adipocytes. Nat Commun.

[29] Lee SM, Jang TH, Park HH (2013). Molecular basis for homo-dimerization of the CIDE domain revealed by the crystal structure of the CIDE-N domain of FSP27. Biochem Biophys Res Commun.

[30] Choi JY (2017). CIDE domains form functionally important higher-order assemblies for DNA fragmentation. Proc Natl Acad Sci USA.

[31] Liu X, Zou H, Widlak P, Garrard W, Wang X (1999). Activation of the apoptotic endonuclease DFF40 (caspase-activated DNase or nuclease). Oligomerization and direct interaction with histone H1. J Biol Chem.

[32] Holm L, Sander C (1995). Dali: a network tool for protein structure comparison. Trends Biochem. Sci..

[33] Minor W, Cymborowski M, Otwinowski Z, Chruszcz M (2006). HKL-3000: the integration of data reduction and structure solution–from diffraction images to an initial model in minutes. Acta Crystallogr D Biol Crystallogr.

[34] McCoy AJ (2007). Solving structures of protein complexes by molecular replacement with Phaser. Acta Crystallogr D Biol Crystallogr.

[35] Emsley P, Cowtan K (2004). Coot: model-building tools for molecular graphics. Acta Crystallogr D Biol Crystallogr.

[36] Vagin AA (2004). REFMAC5 dictionary: organization of prior chemical knowledge and guidelines for its use. Acta Crystallogr D Biol Crystallogr.

[37] Laskowski, R. A., MacArthur, M. W., Moss, D. S. & Thornton, J. M. PROCHECK: a program to check the stereochemical quality of protein structures. *J. Appl. Cryst*. **26** (1993).

[38] DeLano WL, Lam JW (2005). PyMOL: A communications tool for computational models. Abstracts of Papers of the American Chemical Society.

[39] Chen YH, Yang JT, Martinez HM (1972). Determination of the secondary structures of proteins by circular dichroism and optical rotatory dispersion. Biochemistry.

[40] Peisley, A., Wu, B., Xu, H., Chen, Z. J. & Hur, S. Structural basis for ubiquitin-mediated antiviral signal activation by RIG-I. *Nature* (2014). 10.1038/nature13140 PMC6136653 24590070

[41] Ding H (2005). Solution structure of human SUMO-3 C47S and its binding surface for Ubc9. Biochemistry.

[42] Mishra SK (2011). Role of the ubiquitin-like protein Hub1 in splice-site usage and alternative splicing. Nature.

