# Supplementary material for: Crystal structure and mutation analysis revealed that DREP2 CIDE forms a filament-like structure with features differing from those of DREP4 CIDE
Source: Sci Rep. 2018 Dec 13;8:17810. doi: 10.1038/s41598-018-36253-y (PMC6292858; doi:10.1038/s41598-018-36253-y)
Supplement: Supplementary file 1 — Supplementary Figure [file 41598_2018_36253_MOESM1_ESM.pdf]

MICROBIOLOGY, BIOCHEMISTRY, STRUCTURAL BIOLOGY

Crystal structure and mutation analysis revealed that DREP2 CIDE forms a filament-like structure with features differing from those of DREP4 CIDE

Hyun Ji Ha and Hyun Ho Park*

College of Pharmacy, Chung-Ang University, Seoul 06974, Republic of Korea

*Corresponding author: Hyun Ho Park, Phone: +82-2-820-5930. Fax: +82-53-810-4516. E-mail: xrayleox@cau.ac.kr

Supplementary Figure S1

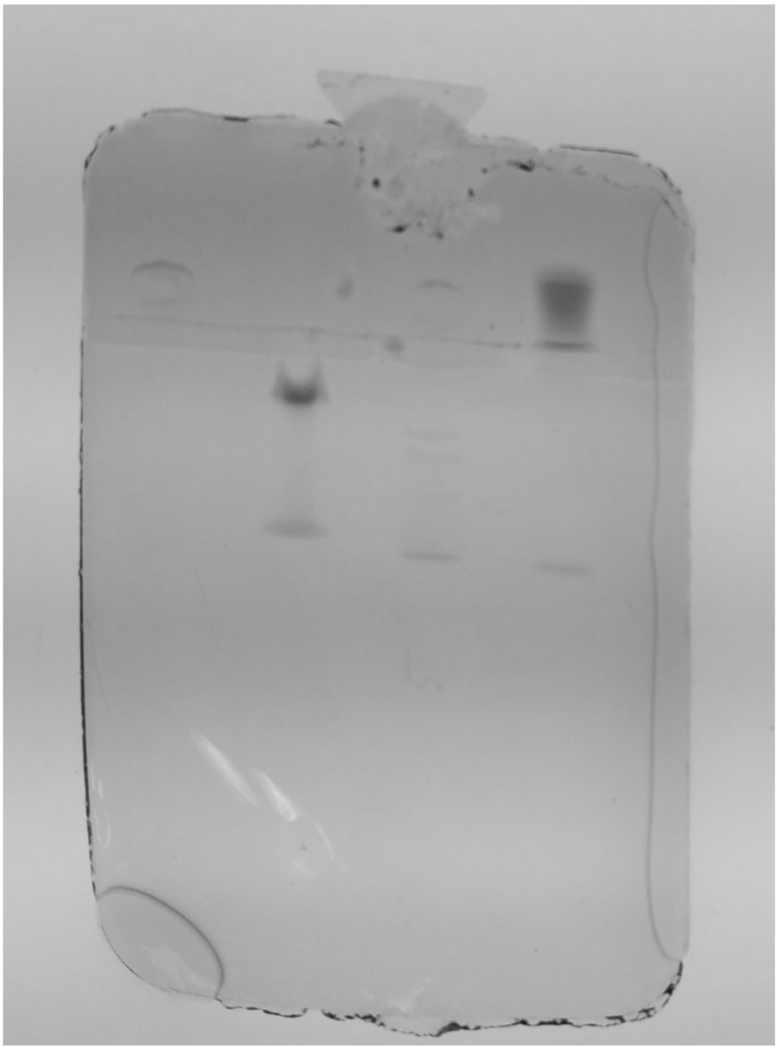

Figure S1. Full-length, uncropped gel for Figure 1C.
